# Supplementary figures and images for: Overcoming extrapolation challenges of deep learning by incorporating physics in protein sequence-function modeling
Source: PLoS Comput Biol. 2026 Mar 25;22(3):e1013728. doi: 10.1371/journal.pcbi.1013728 (PMC13048498; doi:10.1371/journal.pcbi.1013728)

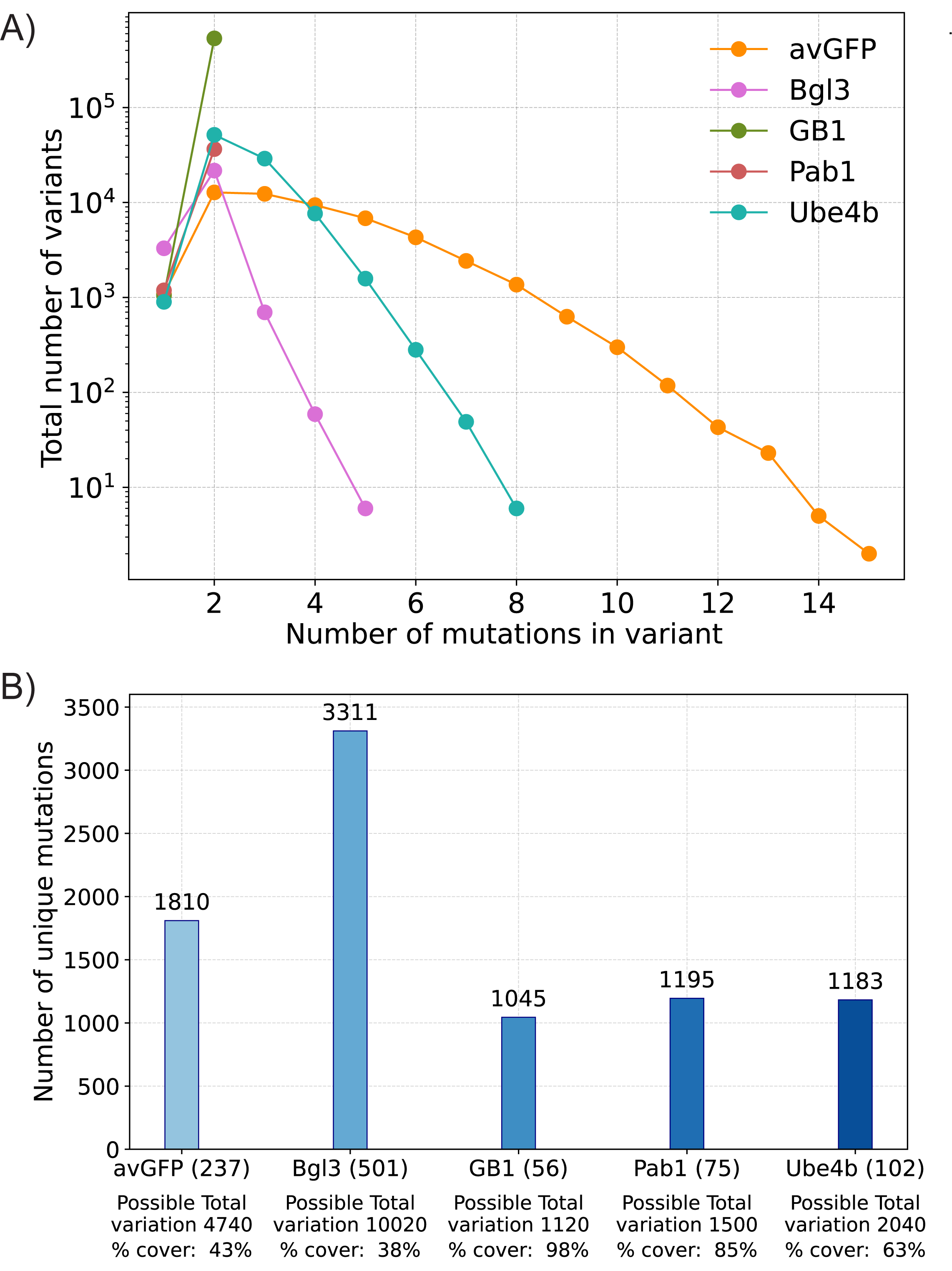

Supplement: S1 Fig — A) Distribution of various containing different number of mutations in each of the five datasets. B) Number of unique mutations sampled for each protein. The coverage percentage, listed under the x-axis, is defined as the number of unique mutations (+ wildtype) divided by the total possible number (20 x sequence length). The sequence length of each protein is given in parathesis next to the protein name. (TIF) [file pcbi.1013728.s001.tif]

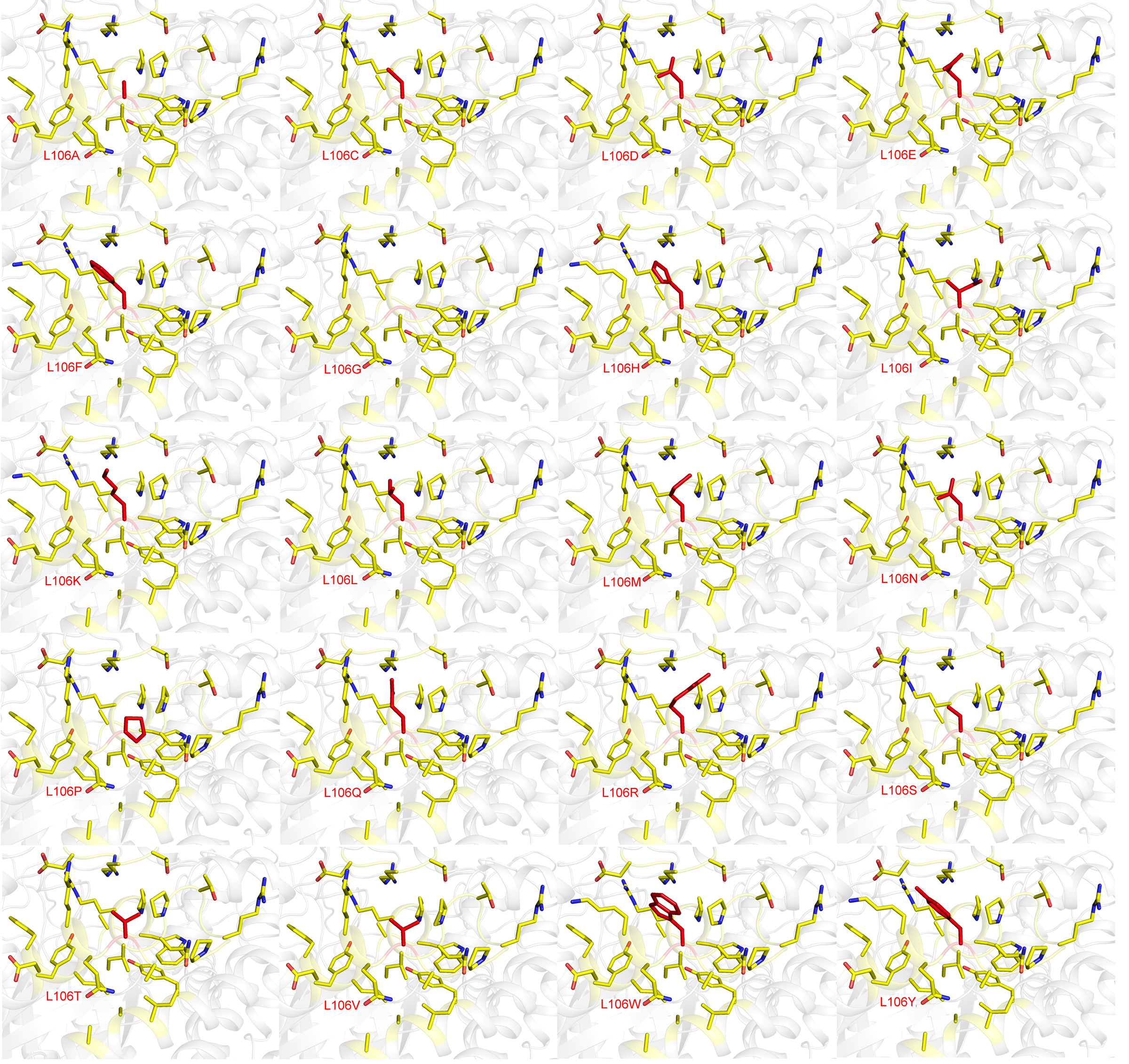

Supplement: S3 Fig — The protein backbone is shown in white cartoon and all residues within the repacking radius are represented as yellow sticks with residue L106 highlighted in red. Elements are colored as follows: C (yellow), N (blue), O (red), and S (orange). (TIF) [file pcbi.1013728.s002.tif]

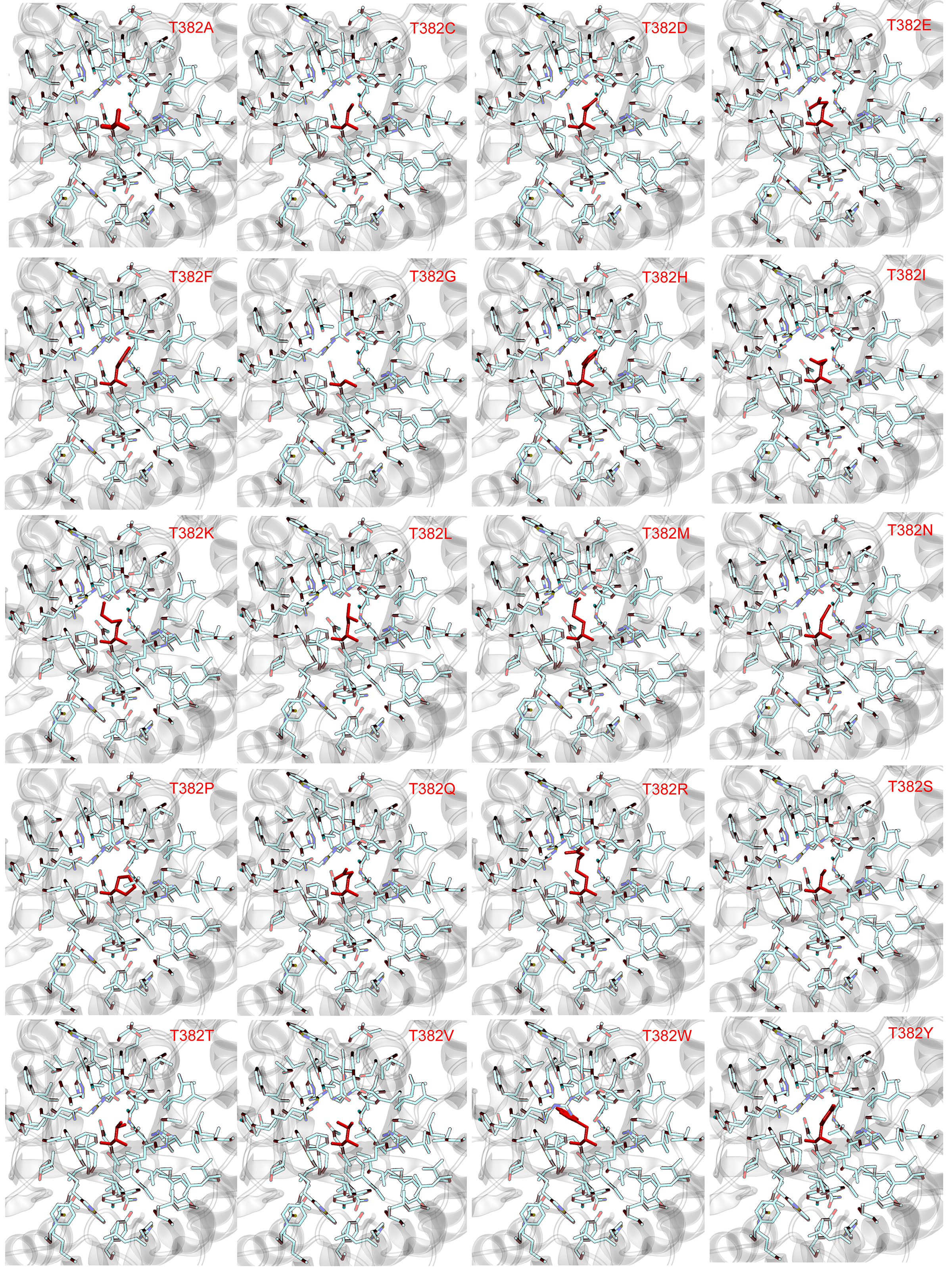

Supplement: S4 Fig — The protein backbone is shown in white cartoon and all residues within the repacking radius are represented as cyan sticks with T382 highlighted in red. Elements are colored as follows: C (cyan), N (blue), O (red), and S (yellow). (TIF) [file pcbi.1013728.s003.tif]

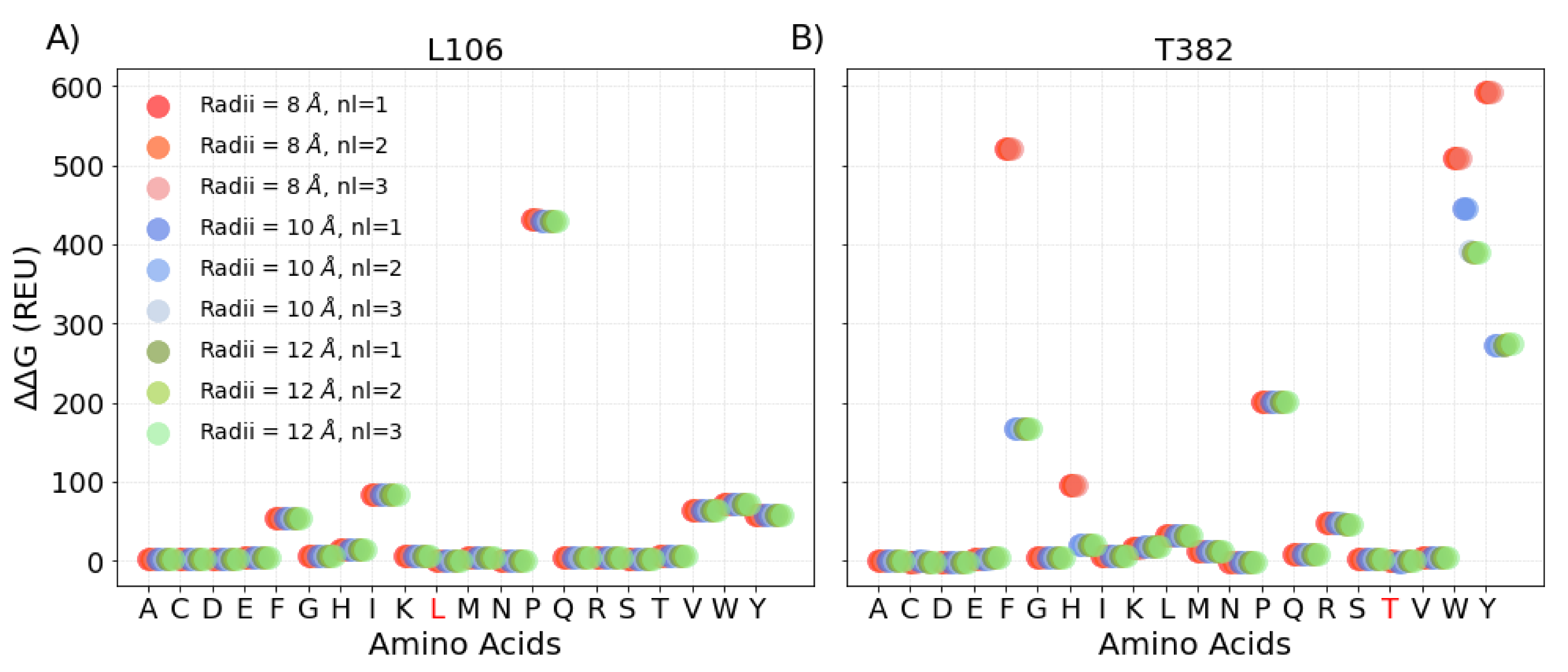

Supplement: S5 Fig — The ΔΔG (in REU) values for the 20 amino acid substitution was calculated for solvent exposed L106 (A) and buried T382 (B). (TIF) [file pcbi.1013728.s004.tif]

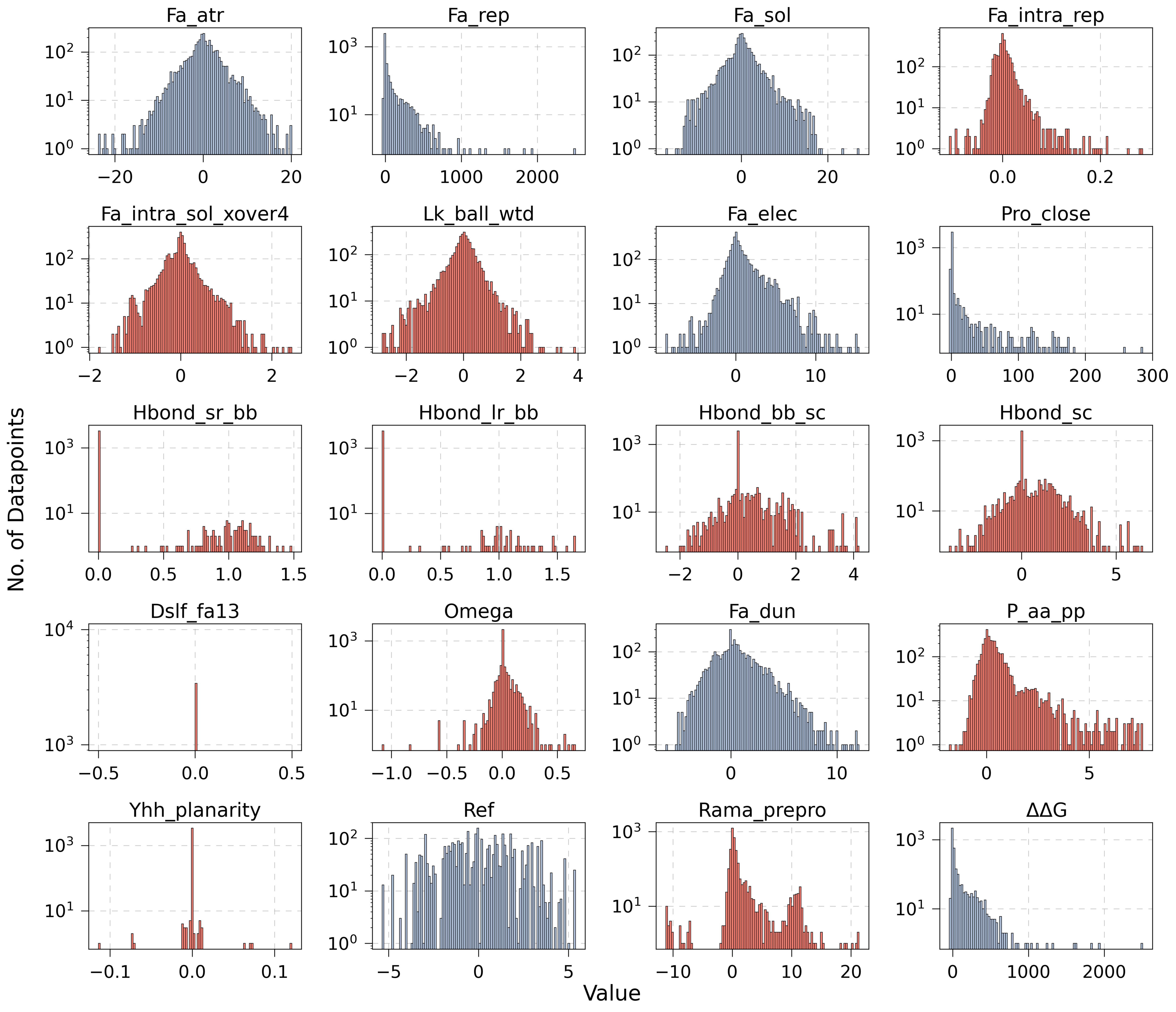

Supplement: S6 Fig — The blue bar plots indicate term used as the input features and red bar plots indicate terms discarded as the input features. (TIF) [file pcbi.1013728.s005.tif]

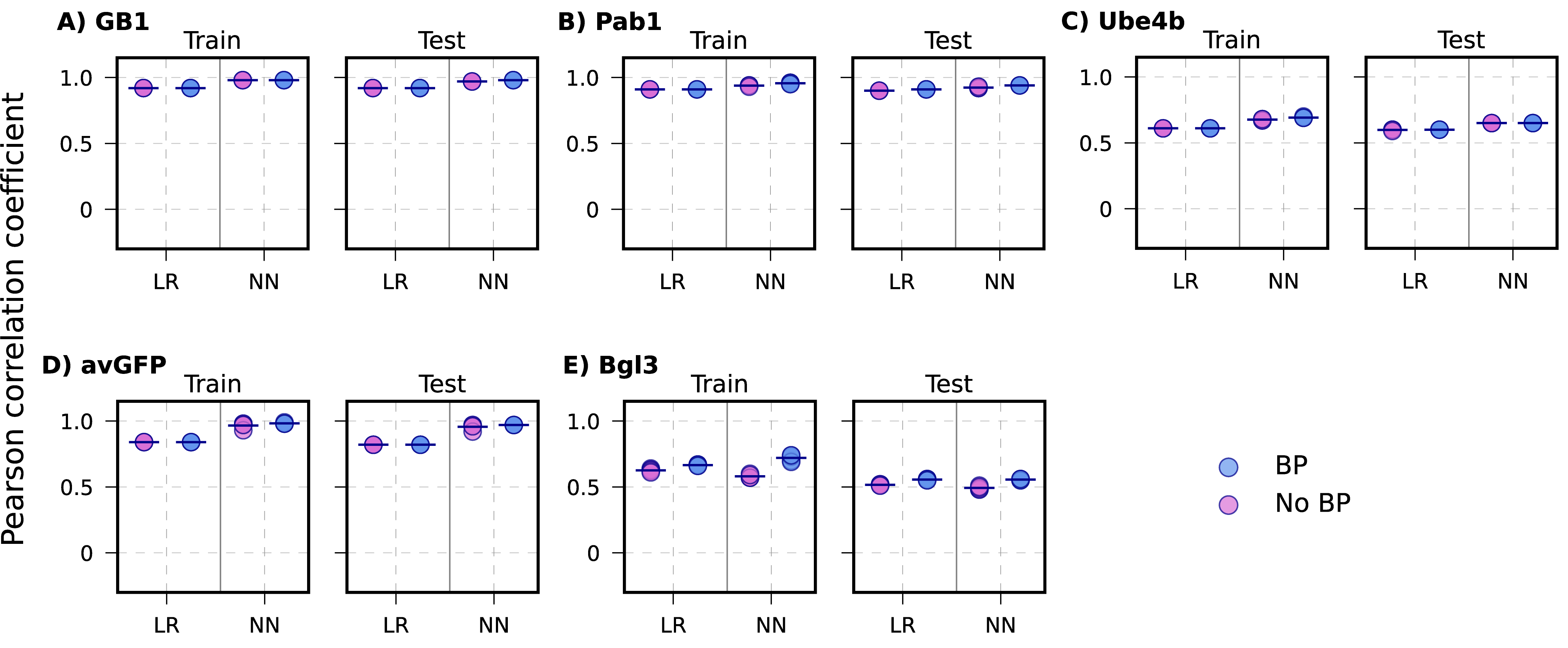

Supplement: S7 Fig — The 5 proteins are arranged from the largest to smallest sequence space coverage: A) GB1, B) Pab1, C) Ube4b, D) avGFP, and E) Bgl3 for with biophysics-based mode (BP) and without-biophysics model (No BP). Each point indicates one of the 5 random train/test splits, and the mean of the 5 replicates are marked using black lines. (TIF) [file pcbi.1013728.s006.tif]

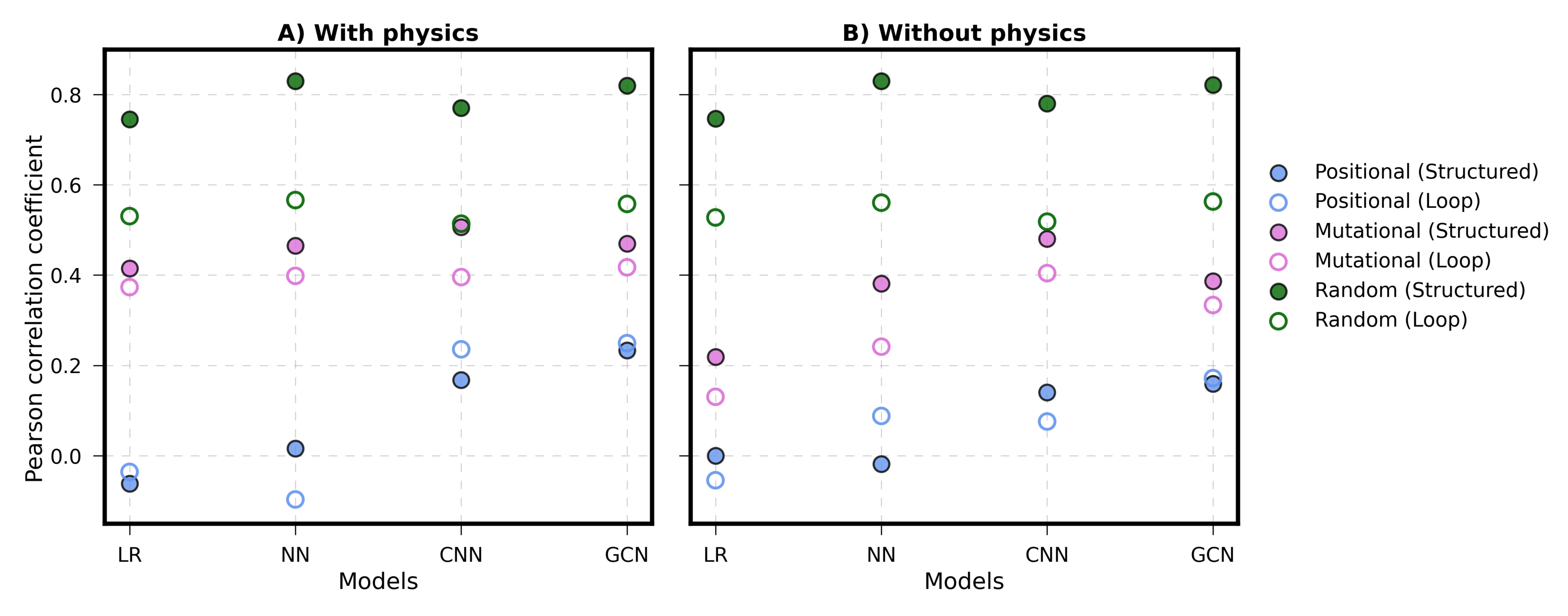

Supplement: S8 Fig — A) Results obtained with BP model and B) Results obtained without BP model for 3 splitting schemes. (TIF) [file pcbi.1013728.s007.tif]

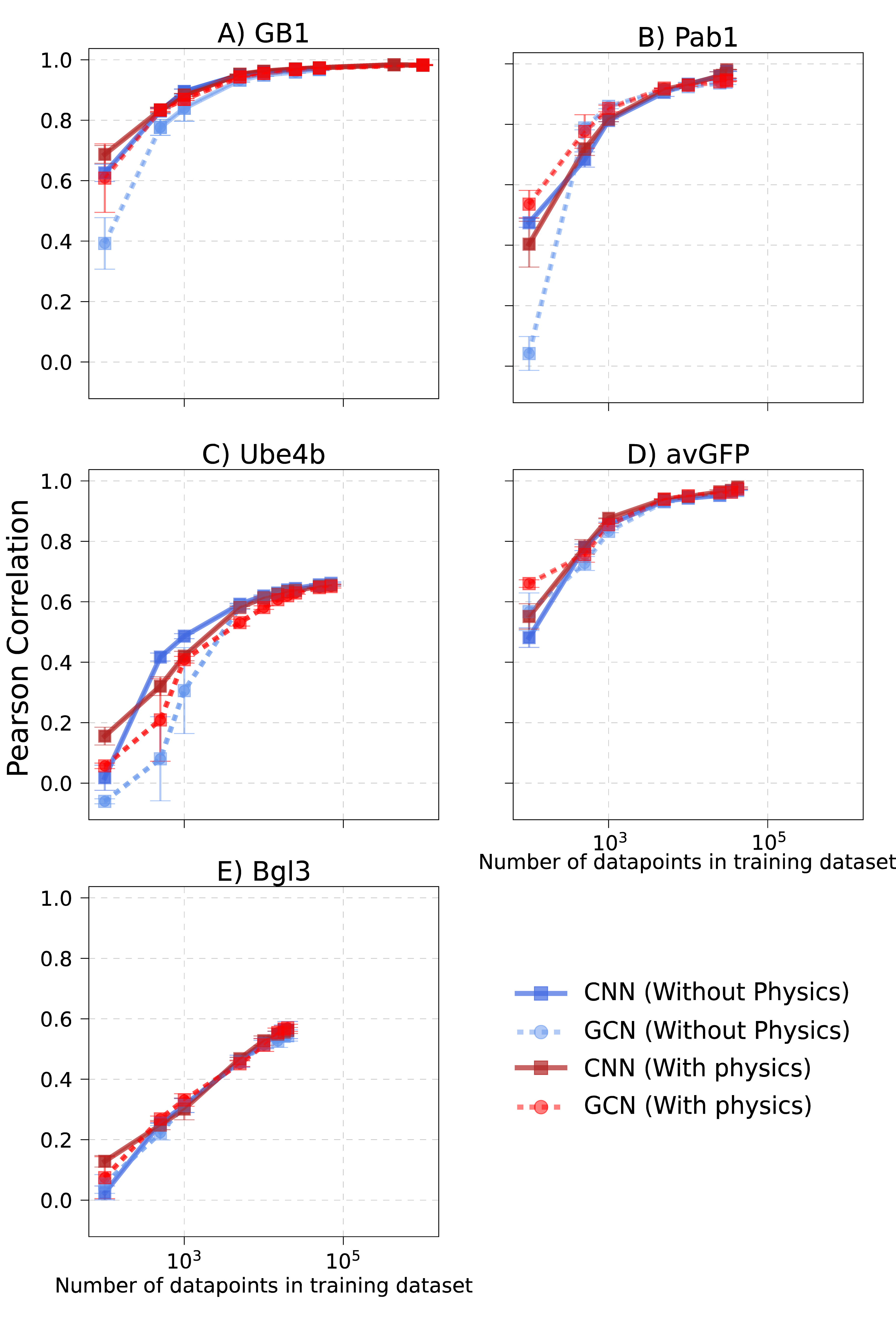

Supplement: S9 Fig — The training dataset was increased from 100 datapoints to 0.9 fraction of the total dataset of each protein. The solid line indicates CNN, and dashed line indicates GCN model. (TIF) [file pcbi.1013728.s008.tif]

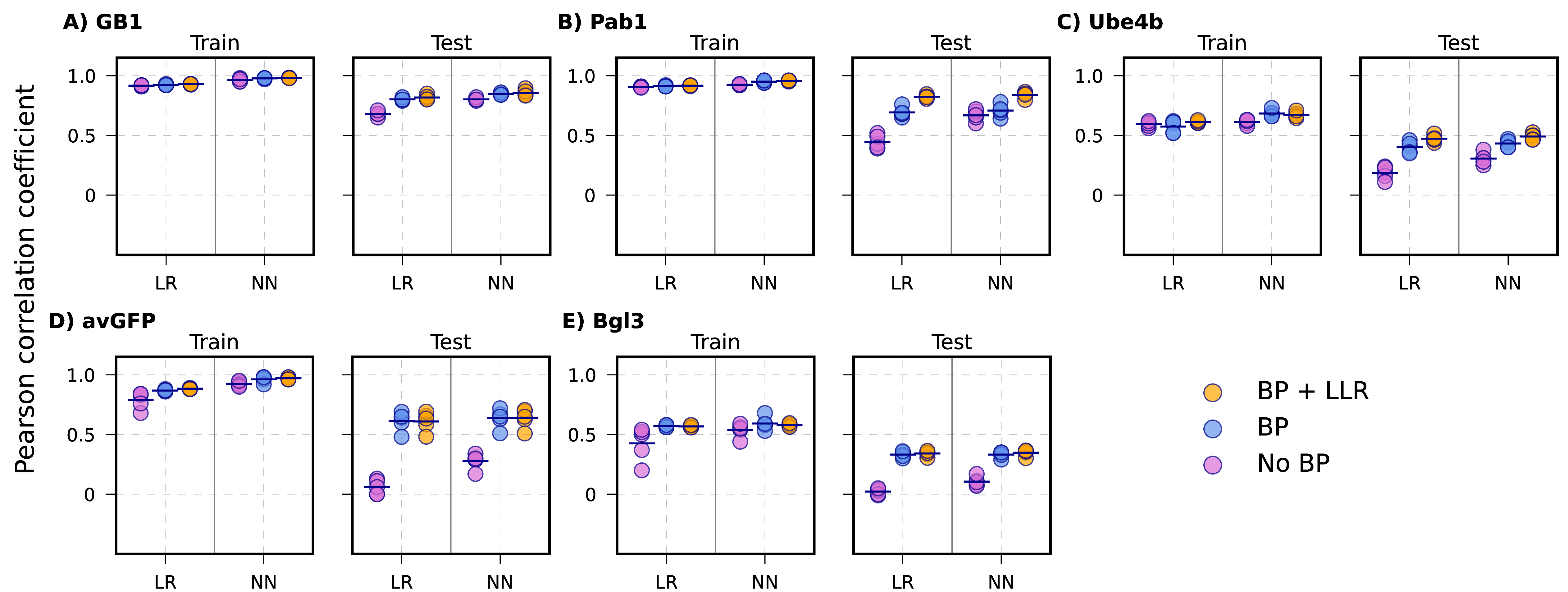

Supplement: S10 Fig — The 5 proteins are arranged from the largest to smallest sequence space coverage: A) GB1, B) Pab1, C) Ube4b, D) avGFP, and E) Bgl3. Each point indicates one of the 5 random train/test splits, and the mean of the 5 replicates are marked using black lines. (TIF) [file pcbi.1013728.s009.tif]

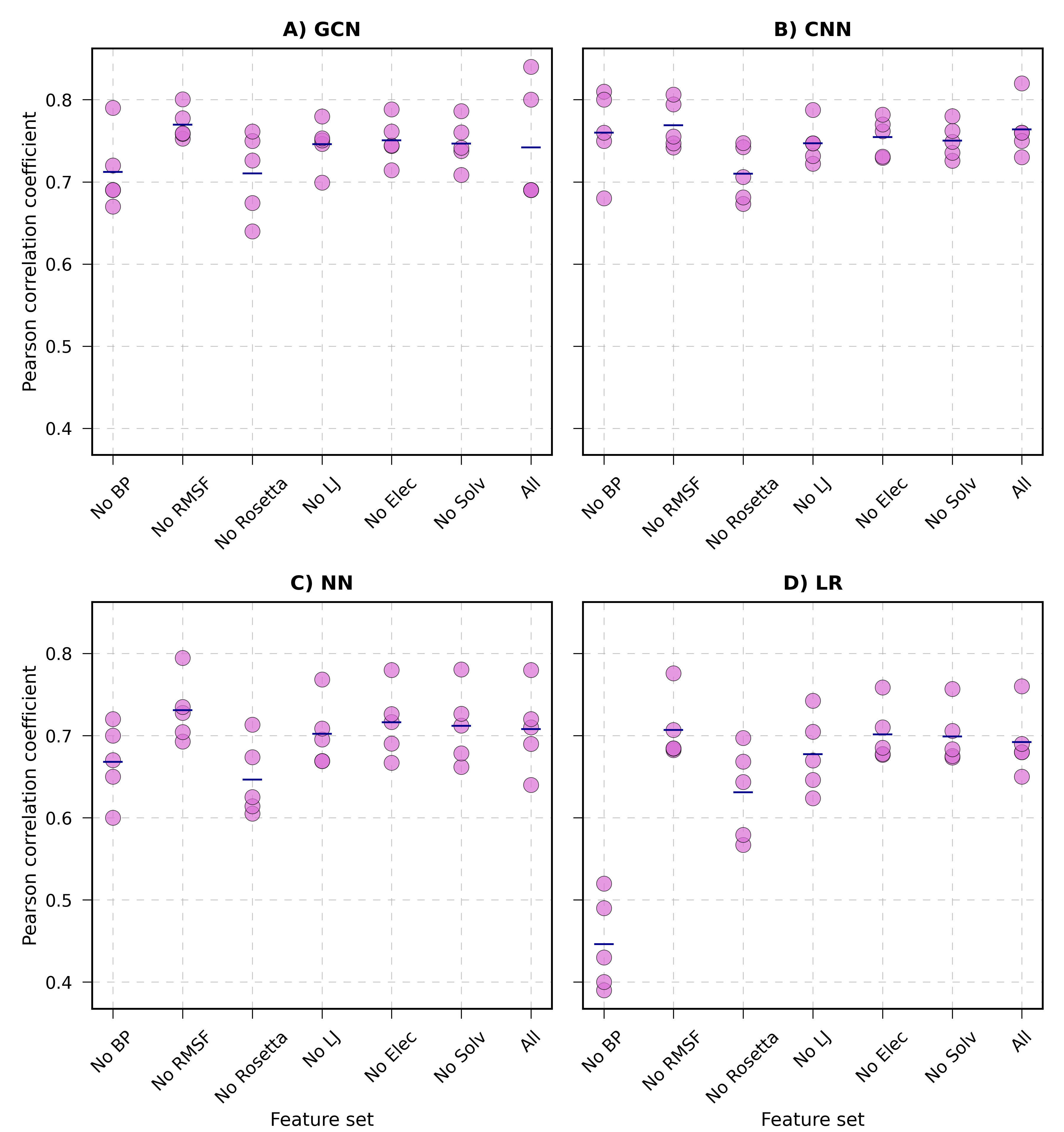

Supplement: S11 Fig — The ablation study is done for the four different models A) GCN, B) CNN, C) NN, and D) LR. Each point represents one of five random train/test splits, and the mean across the five replicates is indicated by black lines. Models were trained using different feature subsets: no biophysics (No BP), no RMSF, no Rosetta, no Lennard–Jones and no ΔΔG (No LJ), no electrostatics and no ΔΔG (No Elec), no solvation and no ΔΔG (No Solv), and all biophysical features included (All). (TIF) [file pcbi.1013728.s010.tif]

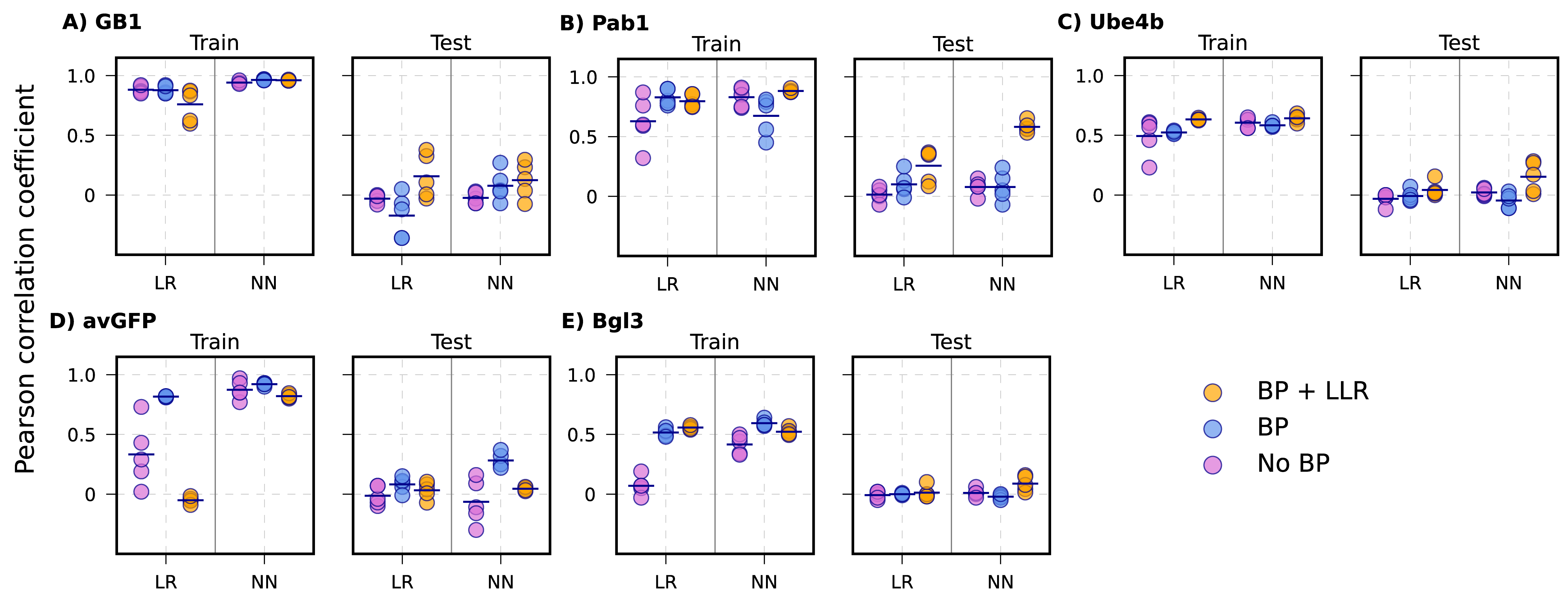

Supplement: S12 Fig — The 5 proteins are arranged from the largest to smallest sequence space coverage. A) GB1, B) Ube4b, C) avGFP, D) Pab1, and E) Bgl3. Each point indicates one of the 5 random train/test splits, and the mean of the 5 replicates are marked using black lines. (TIF) [file pcbi.1013728.s011.tif]

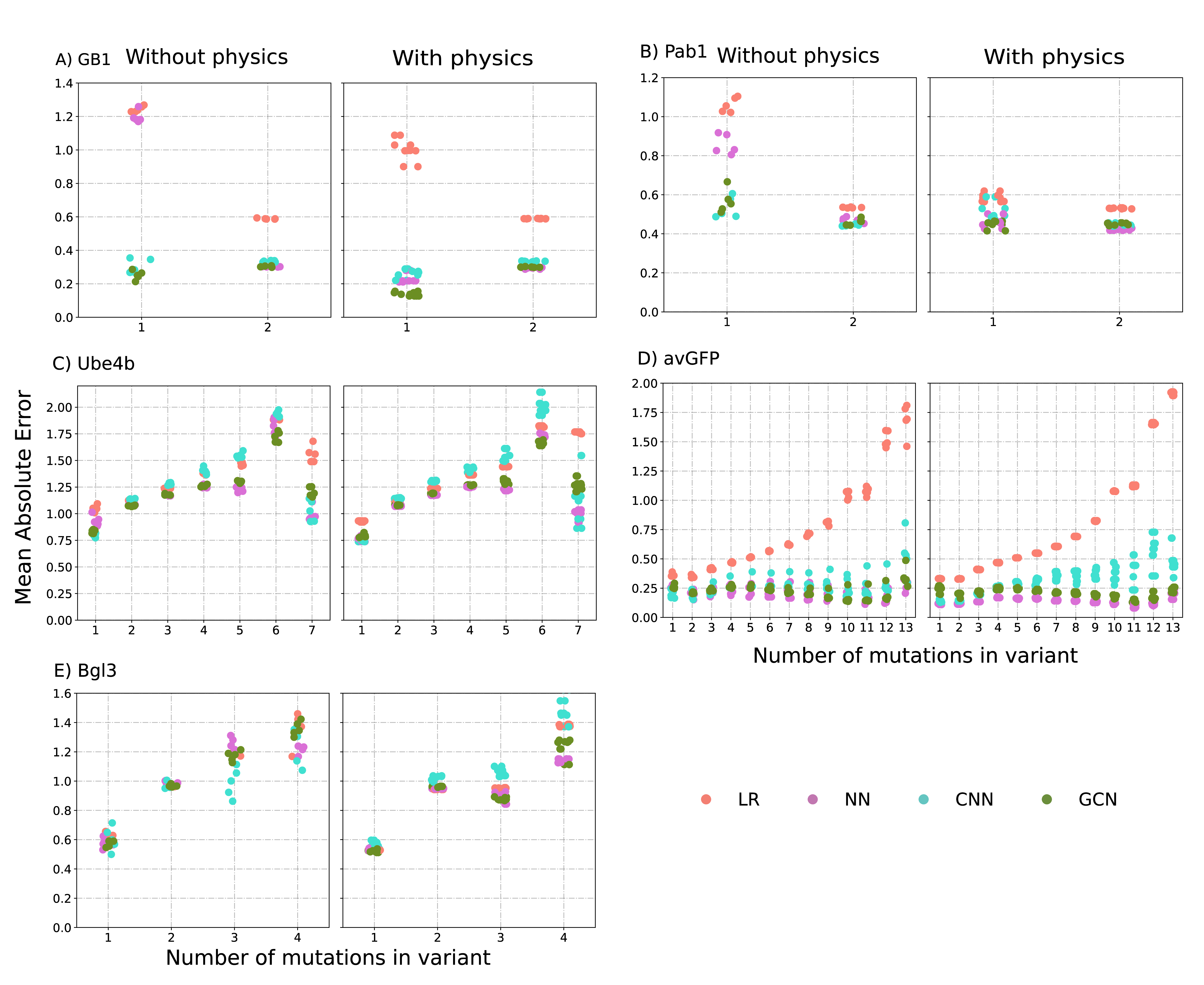

Supplement: S13 Fig — Each color point represents one of the 5 random train/test splits, for LR (salmon), NN (orchid), CNN (turquoise) and GCN (green) models. (TIF) [file pcbi.1013728.s012.tif]

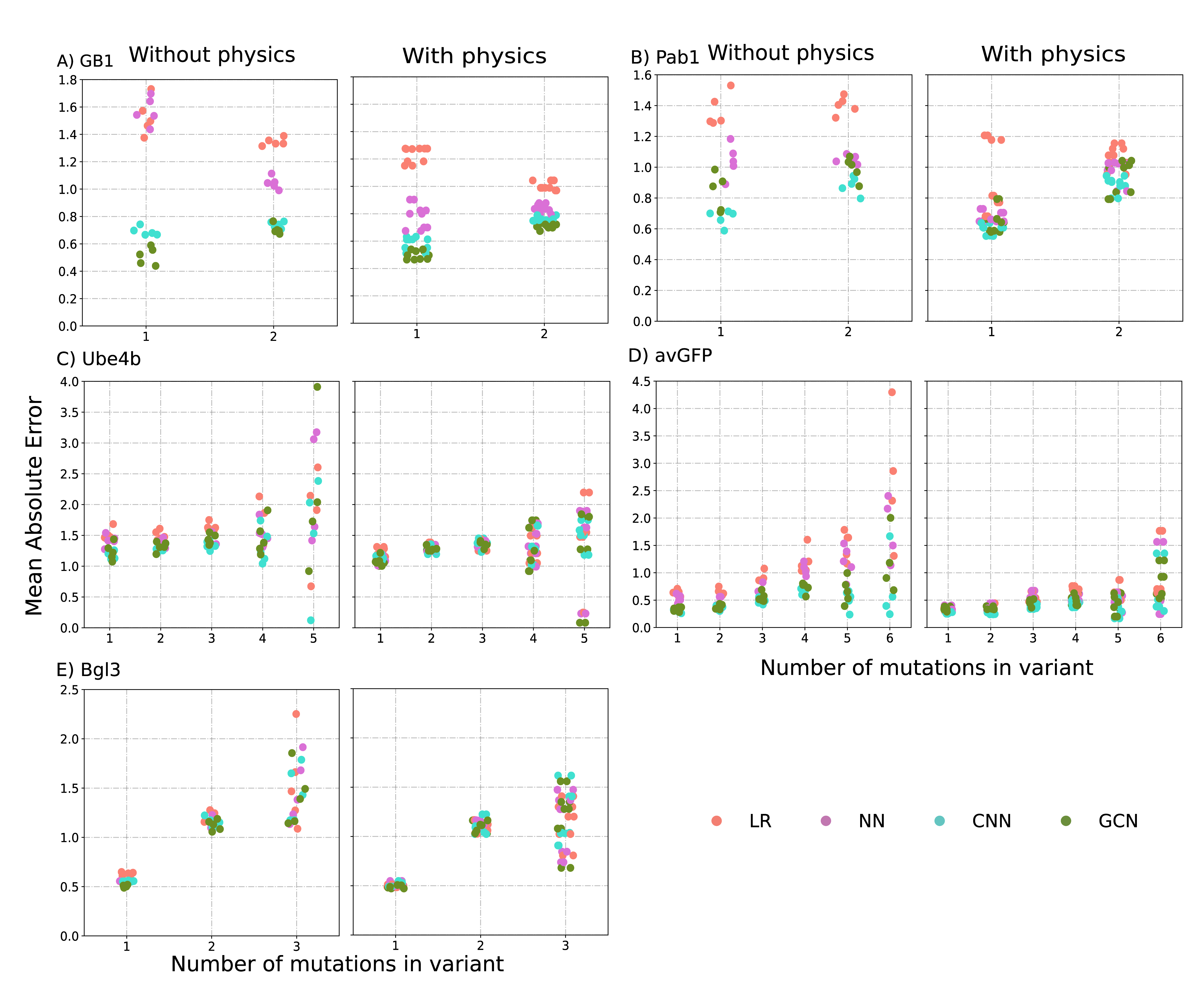

Supplement: S14 Fig — Each color point represents one of the 5 random splits for LR (salmon), NN (orchid), CNN (turquoise) and GCN (green) model. (TIF) [file pcbi.1013728.s013.tif]

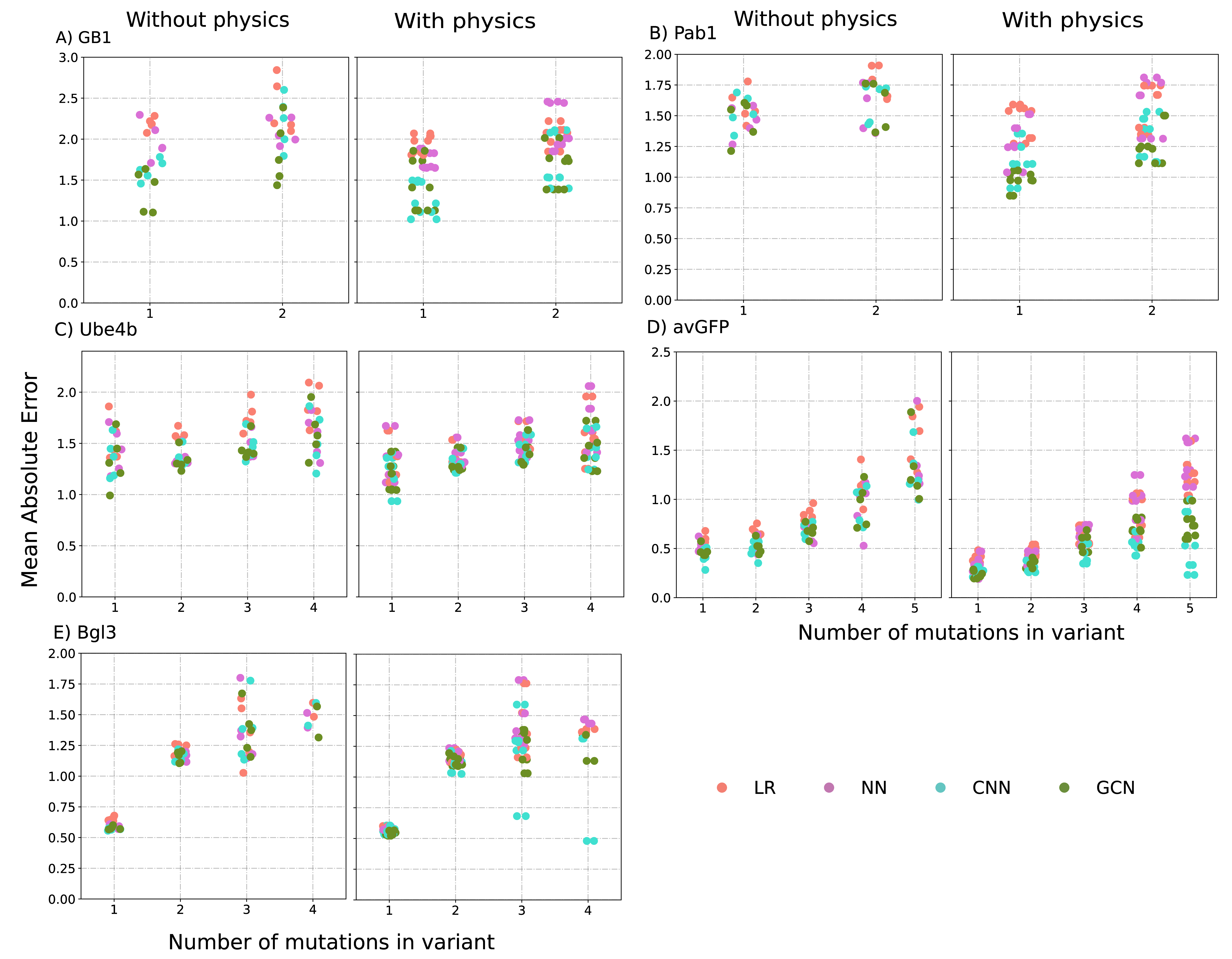

Supplement: S15 Fig — Each color point represents one of the 5 random splits for LR (salmon), NN (orchid), CNN (turquoise) and GCN (green) model. (TIF) [file pcbi.1013728.s014.tif]

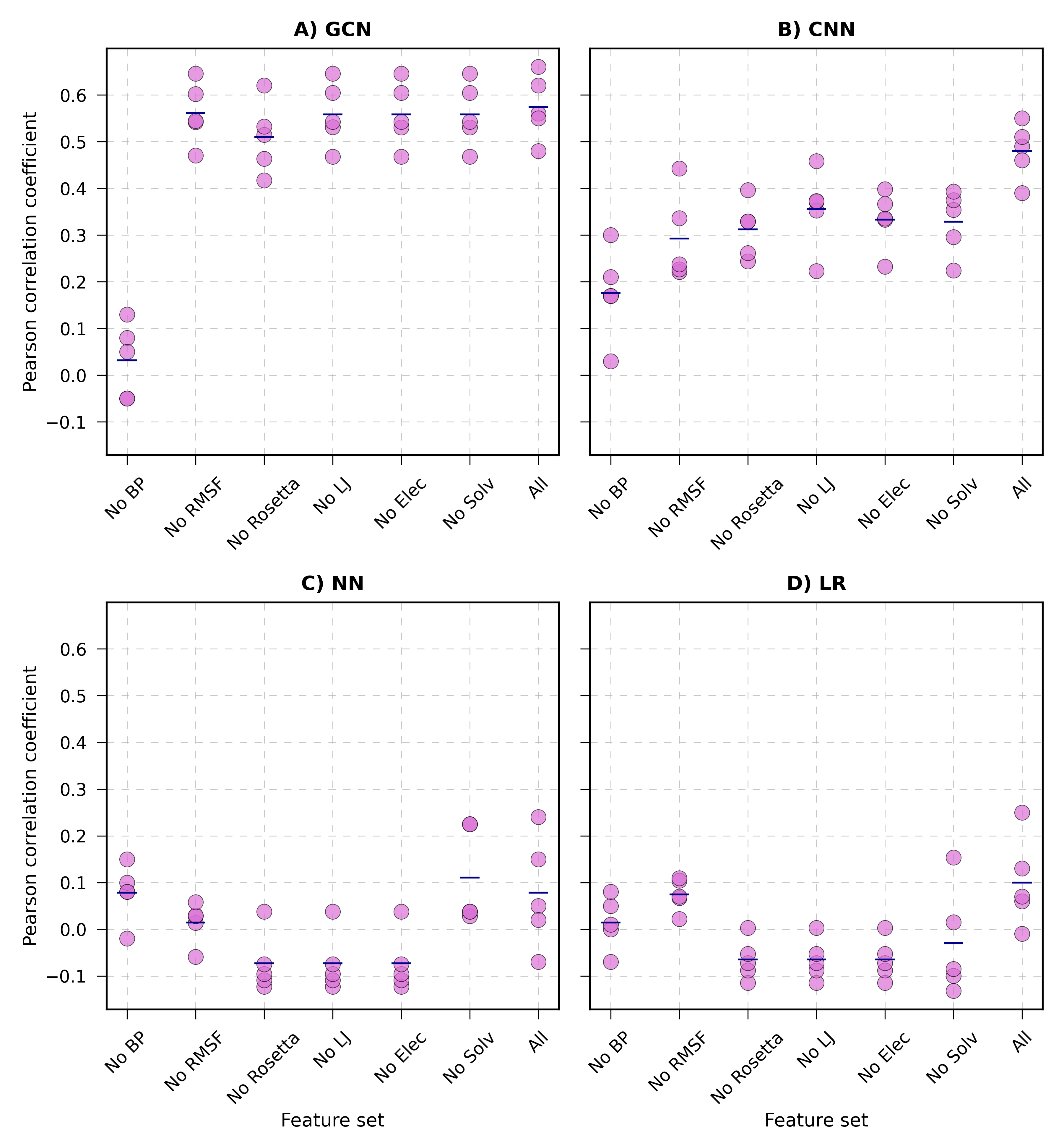

Supplement: S16 Fig — The ablation study is done for the four different models A) GCN, B) CNN, C) NN, and D) LR. Each point represents one of five random train/test splits, and the mean across the five replicates is indicated by black lines. Models were trained using different feature subsets: no biophysics (No BP), no RMSF, no Rosetta, no Lennard–Jones and no ΔΔG (No LJ), no electrostatics and no ΔΔG (No Elec), no solvation and no ΔΔG (No Solv), and all biophysical features included (All). (TIF) [file pcbi.1013728.s015.tif]

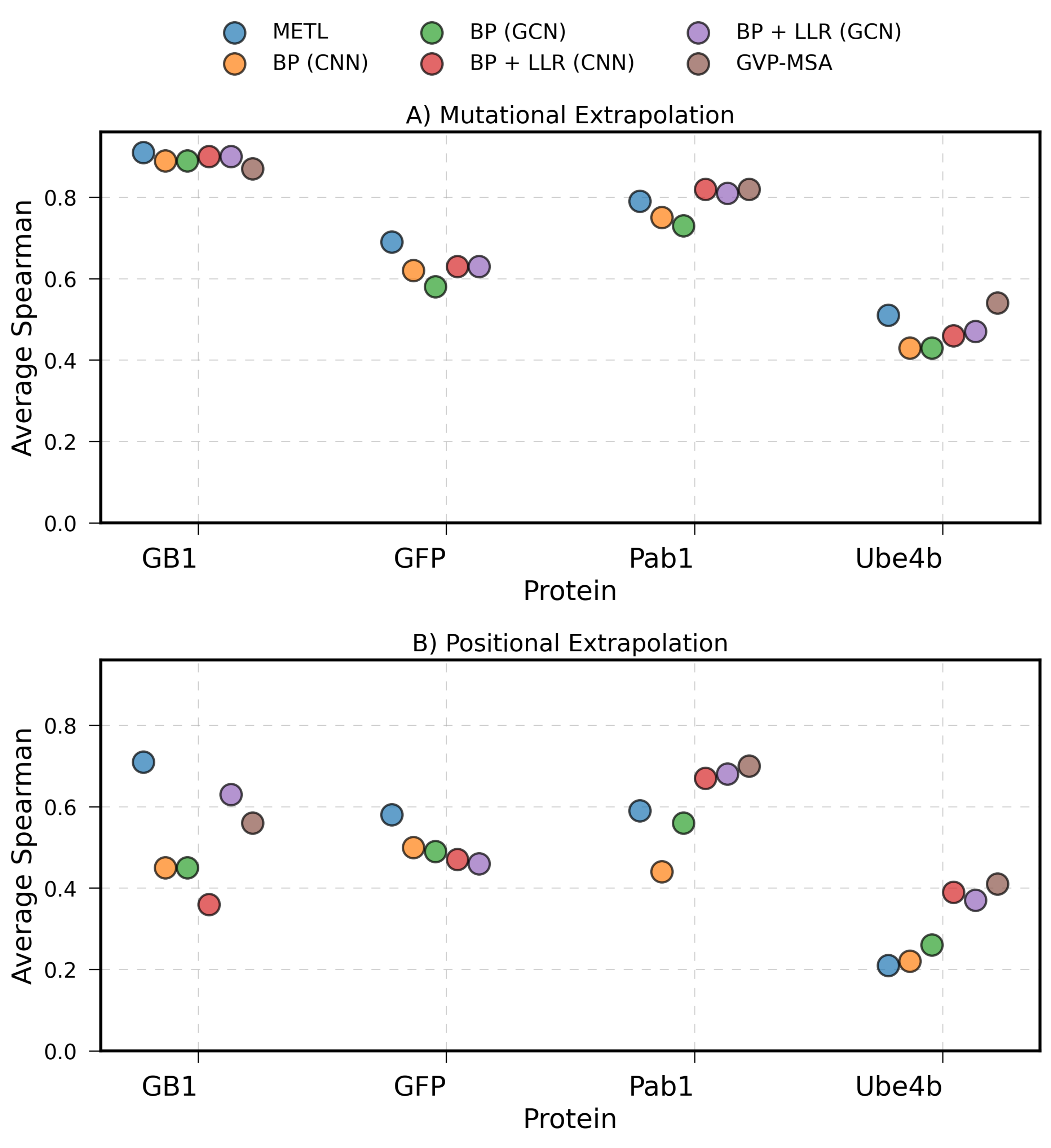

Supplement: S18 Fig — Performance is evaluated using the average Spearman correlation coefficient. Results for METL-local and GVP-MSA (multi-protein) are directly taken from their original publications. For GVP-MSA, only single-mutation variants were considered. (TIF) [file pcbi.1013728.s016.tif]
