# Supplementary material for: Overcoming extrapolation challenges of deep learning by incorporating physics in protein sequence-function modeling
Source: PLoS Comput Biol. 2026 Mar 25;22(3):e1013728. doi: 10.1371/journal.pcbi.1013728 (PMC13048498; doi:10.1371/journal.pcbi.1013728)
Supplement: Attachment — Submitted filename: Response2Reviewers_V1.pdf [file pcbi.1013728.s021.pdf]

Reviewer #1: This manuscript presents a valuable approach to overcoming the extrapolation limitations of deep learning in protein sequence-function modeling by incorporating biophysics-based features (Rosetta energy terms and MD-derived RMSF). The topic is highly relevant to protein engineering and variant effect prediction, and the experimental design using five diverse proteins with varying DMS coverage provides a solid foundation for evaluation. However, several critical issues and gaps need to be addressed to enhance the rigor, generalizability, and depth of the study, as outlined below.

Author responses: We greatly appreciate the constructive comments and support.

1. The current work relies heavily on high-quality experimental PDB structures as initial inputs for simulations. To fully assess the method's applicability in real-world scenarios where experimental structures are scarce, the authors should test the sensitivity of the approach to initial structure quality. Specifically, evaluating performance using computationally predicted structures (e.g., AlphaFold2 models, e**specially those with moderate confidence scores**) would help clarify whether the biophysics-integrated strategy is robust to structural inaccuracies and expand the method's practical utility.

Author response: Thank you for raising an important point. The initial structure quality might influence the biophysics-based features and thus model performance. To evaluate the sensitivity of our approach to initial structure quality, we selected two proteins from our dataset: Pab1 (which is well folded with both helices and sheets as secondary structures) and Ube4b (which has many flexible loops). The AlphaFold2 (AF2) predicted structure for Pab1 exhibits very high pLDDT score, whereas the AF2 predicted structure for Ube4b shows moderate-to-high pLDDT score (see **Figure R1** and **R2** below), thereby allowing us to assess robustness across different levels of structure quality. For both proteins, Rosetta-based features were recalculated using the AF2-predicted structures, and models (LR, NN, CNN, and GCN) were retrained and tested using both positional and mutational splitting schemes.

For Pab1, we trained models using both the original structure (initial) and the AF2-predicted structure. The results indicate that the performance is mostly consistent between models derived from the two structures across all ML models and splits, demonstrating that our approach is robust, especially when high-confidence AF2 structures are used (see **Figure R1** as shown below). The only notable difference is observed for CNN and GCN positional extrapolation, where the AF2 structure-based ML models slightly outperformed those derived from the original PDB structure-based biophysical features.

The ube4b protein, both the AF2 and original structure (initial) biophysics-based models, exhibited similar overall performance (see **Figure R2** shown below), indicating that high-to-moderate-confidence predicted structures do not substantially degrade predictive accuracy. Similar to Pab1, the performance of CNN and GCN models for positional split improved slightly with the AF2-derived structure.

Overall, the new analysis suggests that biophysics-based features remain robust when derived from high and moderate-confidence AF2 predicted structures, supporting the applicability of our approach in settings where experimental structures are unavailable.

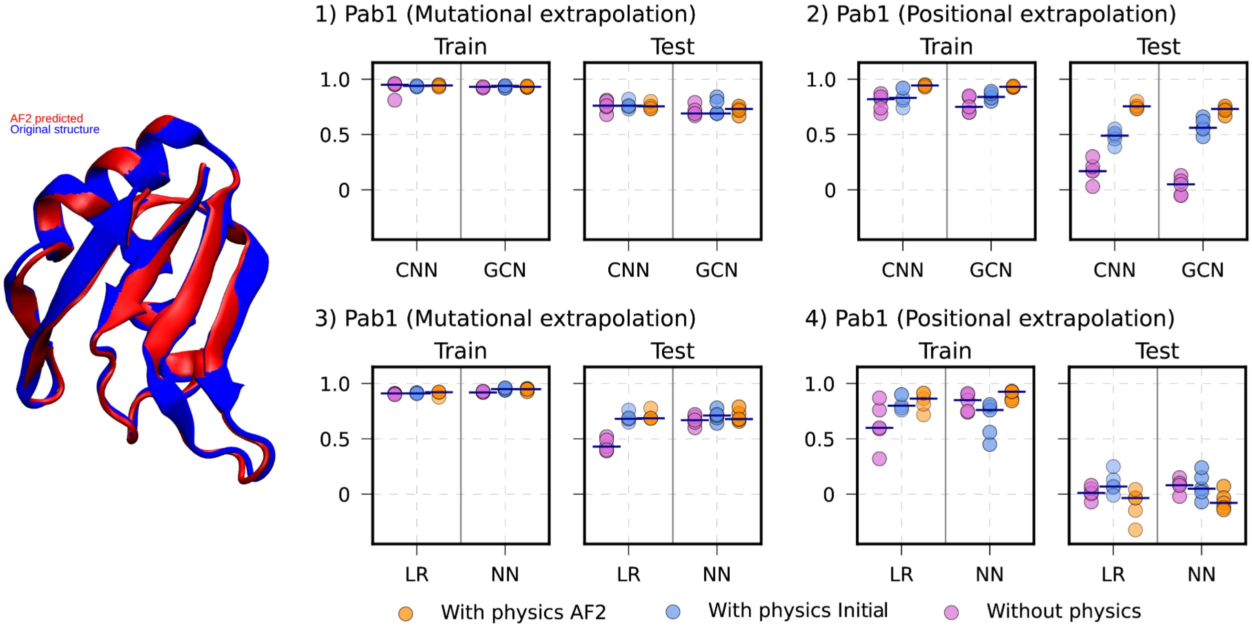

Figure R1: Performance of LR, NN, CNN, and GCN models without biophysics-based features or with biophysics features derived from the original PDB or AF2-predicted structures of protein Pab1 (shown on the left).

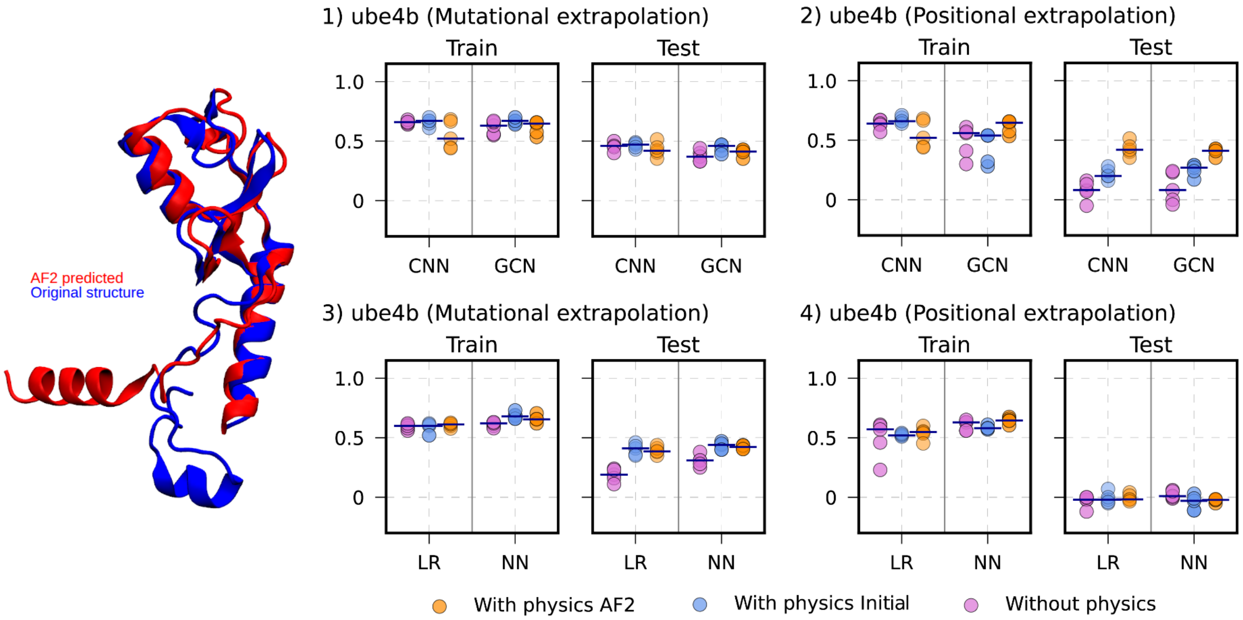

Figure R2: Performance of LR, NN, CNN and GCN models without biophysics-based features or with biophysics features derived from the original PDB or AF2-predicted structures of protein ube4b (shown on the left).

2. While mutational and positional extrapolation are well evaluated, the manuscript lacks an assessment of the model's ability to extrapolate from single variants to higher-order (e.g., double, triple) variant effects. This is a critical use case for VEP tools in protein engineering. The authors should supplement this analysis, including a direct comparison with simple additive models, to demonstrate whether the biophysics-based features capture non-trivial epistatic interactions that improve higher-order variant prediction.

Author response: Thank you for this valuable suggestion. We have now added an explicit evaluation of the model's ability to extrapolate from single-mutation variants to higher-order variants. The results are presented in the main text (**new *Figure 8***) and in the Supplementary Information (**new *Figure S17***).

Overall, we observed that all the models performed very similarly with and without biophysics features. An exception is the avGFP dataset, where all ML models particularly CNN and GCN with biophysics features consistently outperform the counterparts without biophysics features in tests. More generally, extrapolation from single to higher-order variants appears to be a comparatively simple task relative to mutational and positional extrapolation regimes considered in this study, with supervised models performing well across architectures. This observation is consistent with previous findings [Gelman et al., Biophysics-based protein language models for protein engineering, Nature Methods 22, 1868–1879 (2025)] where they also observed additivity in many of the mutational datasets. We further note that the lack of cooperativity in high-order variants also supports our model design that requires only Rosetta energetics of all possible single mutations.

A new section has been added to the revised manuscript discussing these results (**Page 19-20**).

3. The discussion of biophysics-based feature (BP) performance across proteins with different DMS coverage does not account for confounding factors, particularly the inherent variability in the "fitness" metrics measured by DMS across different protein systems. For example, fitness could reflect molecular binding, catalytic activity, or stability—processes that vary in their sequence-function mapping complexity. Failing to disentangle the impact of coverage from that of fitness metric heterogeneity makes it difficult to draw definitive conclusions about BP's effectiveness across diverse biological contexts. The authors should address this by, for instance, stratifying analysis by fitness type or including a control that normalizes for mapping complexity.

Author response: Thank you for raising this important point. We agree that the relationship between the biophysics-based feature and the fitness metric measured by DMS has limited overlap. In this study, the BP features primarily correspond to thermostability, and therefore their correlation with experimentally measured fitness is expected to vary depending on how strongly stability contributes to the fitness metric in DMS. While protein stability is not the sole determinant of fitness, it is an important contributor to many functional phenotypes and can capture meaningful trends even when the mapping between sequence and fitness is indirect.

Importantly, all models in this work are trained and evaluated in a protein-specific manner rather than as a single global model. Therefore, each model learns the sequence-fitness mapping for one protein independently, which eliminates the need for normalization of fitness types in the current study. We acknowledge that a normalization for mapping various fitness would be beneficial in global or transferable models across proteins. A brief discussion has been added to the revised manuscript in the concluding Discussion (see **page 21**).

4. The study would benefit from more rigorous ablation studies to quantify the individual contributions of different BP features. Currently, it is unclear whether specific components (e.g., Rosetta energy terms vs. MD-RMSF, or individual energy sub-terms) drive the observed performance gains. Systematic removal of each feature category and comparison of model performance would clarify which biophysical information is most critical, enhancing the interpretability of the approach and guiding future method optimization.

Author response: Thank you for this valuable suggestion. In response, we performed a systematic ablation study to quantify the individual contributions of different biophysics-based features and to evaluate which components drive the observed performance gains. We conducted the ablation experiments on the Pab1 dataset, which was selected because it contains both structured regions and flexible loop regions, making it suitable for evaluating biophysical contributions as a representative protein. Specifically, we conducted this study for the mutational and positional splitting schemes, as the performance of random splitting was very similar for models with and without biophysical features. The different combinations of biophysics-based features:

1. Excluding RSMF features,
2. Excluding all Rosetta energy terms,
3. Excluding Lennard–Jones terms and ΔΔG,
4. Excluding electrostatic terms and ΔΔG, and
5. Excluding solvation terms and ΔΔG.

The results of the ablation study are provided in the Supplementary Material (**new *Figure S11 and S16*)**. For the mutational splits, we observed that without RMSF model performs slightly better than biophysics-based model. Notably, the LR model was the only architecture for which incorporating BP features led to a substantial improvement compared to without BP model. In this case, all the variant models with biophysics-based features have resulted in improved performance. For the positional split, all the variants outperformed the without BP model, and in CNN, the with BP outperformed all the variants. This suggests that all the features are necessary and have a combined effect rather than an individual and no single BP feature category consistently dominates model performance.

Brief discussions of ablation studies are included in the revised manuscript **pages 17** and **19**.

5. For proteins with high structural flexibility (e.g., Ube4b with long loops), the improvement from BP features is modest. The authors should provide a more in-depth analysis of this limitation—

e.g., whether the current BP features (focused on local interactions and static flexibility) fail to capture global conformational dynamics, and suggest potential modifications (e.g., integrating ensemble-based energy calculations or dynamic correlation features) to address this gap. Addressing these points will strengthen the manuscript's scientific impact and ensure that the proposed approach is thoroughly validated for broader application in protein sequence-function modeling.

Author response: Thank you for this insightful suggestion. Analysis of the MD trajectory reveals a wide range of conformations sampled by protein Ube4b, particularly in the N-terminal segment (residues 1-30) (see **Figure R3** below). Because the Rosetta protocol used to derive the BP features only considers local side chain repacking, it does not capture the effects such large scale conformational changes, which will limit the protocol to provide reliable energetics of mutations in highly flexible regions. We further note that this will remain an extremely difficult task even if more advanced backbone resampling protocols are used.

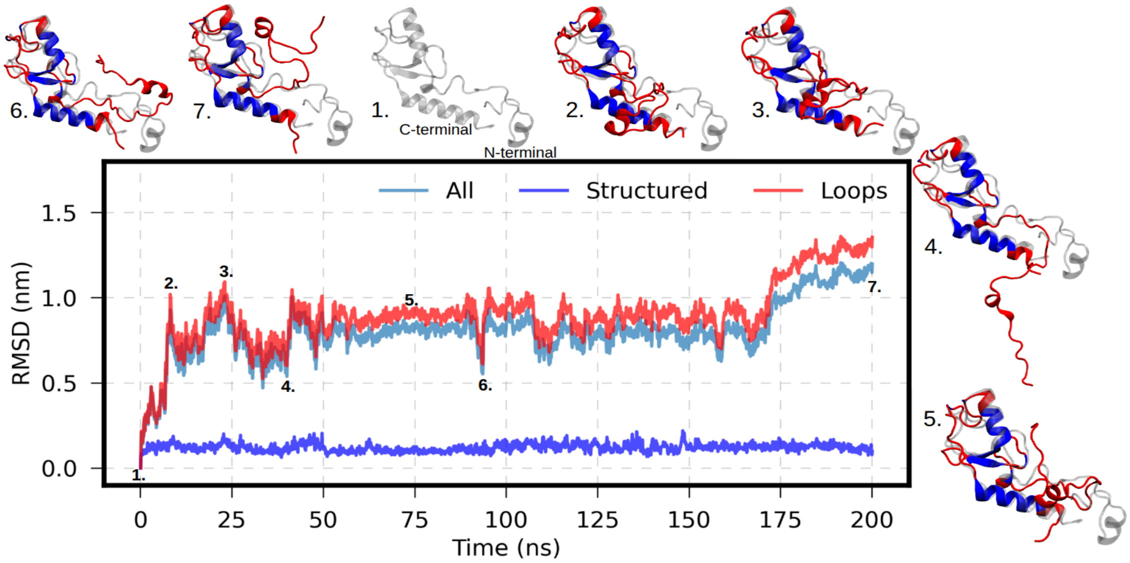

Figure R3. RMSD values as a function of time and representative snapshots of Ube4b.

To further investigate the effects of large conformational dynamics, we performed a comparison between structured and loop regions to determine whether the lower performance for Ube4b (despite higher mutation coverage) originates specifically from flexible loops or is more widespread throughout the protein. This analysis for the random, mutational, and positional splitting scheme is now included in the Results section and provided in the Supplementary Material (**new *Figure S8***). We consistently observed lower correlation values for loop regions across all models compared to structured regions of Ube4b, supporting that the modest improvement from BP features is likely due to the challenges of obtaining accurate mutation energetics for residues within the highly dynamic regions.

A brief discussion has been included in the revised manuscript on **page 14**.

Reviewer #2: In the manuscript under consideration, the authors combined biophysics-based features (Rosetta energetics and MD-derived RMSF) and deep learning architectures (CNN/GCN) to address extrapolation challenges in protein sequence-function modeling. While addressing the data scarcity is a critical goal, I have concerns regarding the novelty of the methodology, the validity of the baselines, and the biophysical assumptions employed. My specific concerns are listed as below.

Author responses: We greatly appreciate the constructive comments of the reviewer.

1) The most critical flaw in this study is the absence of comparison with deep learning methods, specifically Protein Language Models (PLMs) (e.g., ESM-1v, ESM-2) and Inverse Folding Models (e.g., ProteinMPNN), which efficiently leverage structural information without the need for costly MD simulations. These models have demonstrated exceptional zero-shot and few-shot performance on positional extrapolation tasks without requiring explicit physical feature engineering. It is highly probable that a standard fine-tuned ESM-2 model would outperform the proposed method, rendering the complex feature engineering pipeline unnecessary.

Author response: We agree that direct comparisons with protein language models (PLMs) and inverse folding approaches are important for contextualizing performance. However, we have done a comparison with the latest state-of-the-art VEPs such as METL [Gelman et al., Biophysics-based protein language models for protein engineering, Nature Methods 22, 1868–1879 (2025)] and GVP-MSA [Chen et al., Learning protein fitness landscapes with deep mutational scanning data from multiple sources, Cell Systems 14, 706–721 (2023)] models for the positional and regime extrapolation (**new *Figure S18***). Both METL and GVP-MSA have been benchmarked against PLMs and other VEPs such as ESM-2, EVE, ProteinNPT, and DeepSequence for various splitting schemes. In particular, fine-tuned EMS-2 models could indeed provide comparable performance with large training data (Gelman et al, 2025). Therefore, our comparison to METL and GVP-MSA provides meaningful assessment relative to PLM-based methods, without duplicating previously published benchmarks. Our results showed very comparable performance with these models for many of the proteins despite the apparent simplicity.

We also emphasize that the primary goal of this study is to evaluate whether incorporating explicit biophysics-based features can help to overcome data scarcity problems in VEPs. It is likely that the most effective VEPs will include information from both biophysics and sequence evolution as encoded in PLMs. Along this line, we have tested the effect of including log-likelihood ratio (LLR) provided by ESM-2 on mutational and positional extrapolation. The results are included in **revised Figures 6 and 7**, showing additional improvement for some proteins (see **pages 17** and **18**). We note another important advantage of biophysics-informed ML approaches is that they can be readily extended to include effects of post-translational modifications, non-natural amino acids, and ligand binding. We have revised the concluding Discussion to include these points (see **page 21-22**).

2) The strategy of using Wild-Type (WT) RMSF profiles to represent the dynamics of mutant variants is methodologically questionable, particularly for the "extrapolation" tasks the authors aim

to solve. The mutations, especially multi-point ones or those exhibiting epistasis, often significantly alter the protein's conformational ensemble and flexibility. Using WT dynamics as a static feature for all variants ignores the very physical changes that drive functional differences. Therefore, the "physics" incorporated here is an approximation that may not hold for the most challenging prediction targets (i.e., those with significant structural deviations from WT).

Author response: We agree that some mutations could lead to nontrivial effects on protein structure and dynamics and that the effects of these mutations would be extremely difficult to predict (at least for any structural/physics-based approaches). Indeed, this is an approximation that may fail for the most challenging targets. In this work, the RMSF feature mainly provides the expected flexibility. Our ablation studies (**new Figures S11** and **S16, also see response to Reviewer 1 point #4**) suggest that RMSF generally contributes positively to the model performance, suggesting that the RMSF profile derived from WT proteins is a viable feature. Nonetheless, we have included a brief discussion of the caveat of using WT RMSF profile in the revised Method section (see **page 8**).

3) The technical contribution appears to be primarily feature engineering—simply concatenating Rosetta energy terms and RMSF values into the input layer of standard CNN/GCN architectures. In the Discussion section, the authors mentioned that combining these features as input of the Transformer-based architectures could improve performance. Given that Transformer-based architectures are now standard, sticking to older architectures (CNN/GCN) without a compelling justification limits the impact and relevance of the study.

Author response: We agree that our work involves limited technical contributions. The aim of this study is to test if incorporating biophysics-based features could provide a simple but effective strategy for overcoming the data scarcity problem in training protein-specific VEPs. To this end, we deliberately focused on comparatively simpler models (LR, NN, CNN, and GCN) that have been recently evaluated by Gelman at al (PNAS 2021) to assess the impact of incorporating biophysics-based features. We have also compared our approach against two state-of-the-art transformer-based methods (provided in a **new *Figure S18***), including METL (Gelman et al., Nature Methods 2025) and GVP-MSA (Chen et al., Cell Systems 2023). The results suggest that these relatively simple models perform comparably to these transformer-based models. The simplicity of our approach could thus be considered an advantage. Importantly, as noted above, the biophysics-based nature allow our approach to be readily extended to include prediction of effects of post-translational modifications, non-natural amino acids, ligand binding and others. A brief discussion has been added to the concluding Discussion (see **pages 21-22**).

4) While the authors claim "significant enhancement," the absolute performance in challenging cases remains poor. For example, in the Ube4b positional extrapolation task, the Pearson correlation only reaches ~0.2-0.25 even with biophysical features. This suggests that the added physical features fail to capture the necessary signal for flexible proteins. The trade-off between the high computational cost (running MD and Rosetta for feature generation) and the marginal performance gain in difficult cases is not well-justified.

Author response: We agree that the performance of Ube4b with biophysics model does not significantly increased compare to the without biophysics models. However, if we compare the other state-of-the-art models, we can see that the performance is very comparable for the positional extrapolation (Our Spearman correlation coefficient: ~0.25 , METL Spearman correlation coefficient: ~0.2, GVP-MSA Spearman correlation coefficient for the global model: ~0.4 and protein-specific model: ~0.25).

To investigate the causation of lower performance, we performed a comparison between structured and loop regions to determine whether the lower performance (despite higher mutation coverage) originates specifically from flexible loops or is independent. This analysis for the random, mutational, and positional splitting scheme is now included in the Results section and provided in the Supplementary Material (***Figure S8***). We consistently observed lower correlation values for loop regions across all models compared to structured regions, supporting that the modest improvement from BP features is primarily driven by their limited ability to capture global conformational dynamics.

We also want to note that the overall workflow in this work is actually very efficient, mainly requiring only sequence length x 19 Rosetta rotamer repacking evaluations. The RMSF profile of the WT protein can be derived from short MD simulations (~100 ns), which can also be completed within hours with implicit solvent. Together, our workflow allows an accurate VEP trained within a few hours given the DMS dataset.

Reviewer #3: This study focuses on the extrapolation challenges faced by deep learning in protein sequence-function modeling. It integrates biophysical features such as Rosetta energy terms and residue root mean square fluctuation (RMSF) derived from molecular dynamics (MD) simulations into models including convolutional neural networks (CNNs) and graph convolutional neural networks (GCNs). This integration effectively enhances the models' predictive performance for unseen mutation types (mutational extrapolation) and mutation positions (positional extrapolation), providing a highly valuable physics-informed solution to address protein variant effect prediction (VEP) problems under data scarcity scenarios. However, the following issues need to be resolved prior to publication.

Author responses: We greatly appreciate the constructive comments of the reviewer.

1. Insufficient verification of parameter robustness for physical feature calculation. The study only optimized two parameters (repacking radius and nloop) for Rosetta's PackRotamerMover protocol, but failed to verify the impact of key parameters such as force field version (REF2015) and side-chain library (Dunbrack) on ΔΔG calculations. Variations in parameter combinations may cause fluctuations in energy features, necessitating supplementary parameter sensitivity analysis.

Author response: Thank you for your comment. In the current experimentation for PackRotamerMover is limited to repacking radii and *nloop* (sidechain repacking iterations), as these parameters directly control the local side-chain sampling that directly influences the ΔΔG calculation. We also evaluated the other commonly used FastRelax protocol [Tyka et al., Alternate States of Proteins Revealed by Detailed Energy Landscape Mapping. Journal of Molecular Biology 405: 607–618 (2011)] and observed that PackRotamerMover provides a significantly higher performance than the FastRelax. In the FastRelax protocol, relaxation of the backbone as well the sidechain results in the noisier energetics, which is likely due to challenges in accurately capturing energetic effects of larger structural relaxation. Whereas in the PackRotamerMover protocol only sidechains within certain radii are allowed to repack, drastically reducing background noise in mutational energetics.

Regarding the Rosetta energy function version, we believe that using the latest Ref2015 score function with Dunbrack rotamer library is the default and most rational choice in Rosetta, as it has been widely validated for protein design and ΔΔG calculation.

2. Questionable sufficiency of MD simulation duration and conformational sampling. A 200-ns MD simulation may lead to inadequate conformational sampling for flexible proteins (e.g., Ube4b with long loops). The study did not calculate the convergence of root mean square deviation (RMSD) or adopt enhanced sampling methods (e.g., metadynamics), making it difficult to accurately characterize the intrinsic flexibility of residues. Convergence verification of simulations should be supplemented.

In addition, only a single 200-ns all-atom MD simulation was performed per protein to obtain residue Cα RMSF values, with no routine ≥3 independent replicate simulations. This risks RMSF distortion due to initial conformation dependence and random thermal motion, introducing non-

specific model errors that undermine physical feature reliability and conclusion robustness. It is recommended to add at least two replicate MD simulations with identical parameters but different initial random seeds, calculate RMSF mean and standard deviation to assess feature variability, optimize simulation strategies (e.g., extended duration or enhanced sampling) if significant RMSF discrepancies exist across replicates, and retrain models using statistically averaged RMSF data to verify feature stability and improve result reproducibility and credibility

Author response: We agree that in general replica simulations should be performed for rigorous evaluation of convergence. However, MD simulations here are only to provide an estimate of the flexibility of the WT protein as captured in the RMSF profile, and short simulations of ~100-ns are generally adequate. For example, we analyze the convergence in RMSF profiles by dividing the trajectories into two 100-ns segments and the results suggest that they are well converged (see **revised Figure S2**). We agree that these simulations are too short to sample large-scale conformational transitions, particularly for flexible regions such as the N-terminal region of Ube4b (see **Figure R3** above). As discussed above in response to **Reviewer #1 Point #5**, the latter represent extremely challenging cases where the major bottleneck to accurate VEP is difficulty in obtaining reliable mutational energetics. We observe clear correlation between VEP performance and the presence of large-scale conformational transitions (e.g., see new **Figure S8**).

3. Lack of rationality for the additivity assumption of multi-mutation energy effects. The model assumes that the energy effects of multi-site mutations are linearly additive across single-point mutations, yet epistasis (nonlinear interactions between mutation sites, including synergistic or antagonistic effects) is prevalent in natural proteins. The study did not evaluate the prediction errors induced by this additivity assumption, and should compare energy differences between the additive model and all-atom calculations for multi-site mutations.

Author response: Thank you for the comment. We would like to first clarify that the proposed model does not assume linear additivity of energy effects across mutations. Instead, features are added independently at each sequence position, without enforcing an additive relationship between single-site contributions. Additionally, we have evaluated the models' ability to extrapolate from single-mutation variants to higher-order variants (**new *Figure 8*** and ***Figure S17***). Please refer to our response to **Reviewer #1 Point #2** above for details. Consistent with previous studies noted in our response above, the lack of cooperativity in high-order variants supports our model design that requires only Rosetta energetics of all possible single mutations (also see the **new section** on **pages 19-20**).

4. Ambiguous graph construction logic for GCN. The GCN constructs contact maps based on Cα/Cβ atom distances, using a threshold of 6 Å for Bgl3 and 7 Å for other proteins, but does not explain the biological rationality of these thresholds. Comparisons of model performance across different distance thresholds (e.g., 5 Å, 8 Å) should be supplemented to validate graph topology optimization.

Author response: Thank you for this comment. The distance threshold used to construct the contact graph in the GCN is treated as a model hyperparameter rather than a biologically fixed

quantity. In our implementation, the threshold was selected empirically to ensure stable training across proteins of different sizes. In particular, Bgl3 is substantially larger (501 residues) than the other proteins in our dataset (approximately 55–240 residues). Using a smaller cutoff (6 Å) for Bgl3 may help avoid overly dense graphs that can arise in large proteins and may hinder effective message passing, whereas a slightly larger cutoff (7 Å) is required to maintain connectivity for smaller proteins. Thus, the choice reflects practical considerations related to protein size and graph sparsity rather than a strict biological interpretation of residue-residue interactions.

5. Only four traditional architectures (LR/NN/CNN/GCN) were employed, without integrating cutting-edge hybrid models in the AIDD field (e.g., CNN-Transformer, GVP-GNN) or attention mechanisms to capture long-range site correlations. This limits the model's ability to address complex dependencies in high-order mutations (e.g., synergistic multi-site mutations).

Author response: We agree that transformer-based and hybrid architectures could be advantageous for capturing long-range dependencies and complex site interactions, particularly in high-order mutation. However, the primary objective of this study is to investigate the contribution of biophysics-based features to predictive performance, rather than to pursue architectural complexity. To this end, we deliberately focused on comparatively simpler models (LR, NN, CNN, and GCN) to assess the impact of incorporating biophysics-based features. Importantly, we compared our approach against state-of-the-art transformer-based methods, including METL and GVA-MSA. Despite relying on comparatively simpler CNN-based architectures, our biophysics models achieve performance that is highly comparable to these transformer-based models. This suggests that high-quality, biophysics-based features can enable even relatively simple models (CNN-based) without extensive pretraining to achieve performance comparable to that of complex, state-of-the-art architectures.

In response to the reviewer's concern regarding higher-order mutations, we have additionally performed an explicit evaluation of the models' ability to extrapolate from single-mutation variants to higher-order variants. These results are now included in the main text (***Figure 8***) and the Supplementary Information (***Figure S17***). Overall, we observe that models with and without biophysics-based features perform similarly across most datasets. Additionally, none of the models has any limitation to capture the complex dependencies in higher-order variants. This observation is consistent with previous findings [Gelman et al., Biophysics-based protein language models for protein engineering, Nature Methods 22, 1868–1879 (2025)], where they also observed minimal cooperativity in many of the mutational datasets. Nonetheless, we agree that transformer-based models could be required to capture the long-range site correlations and address complex dependencies in high-order mutations. A brief discussion has been added to the concluding Discussion on this important note (see **page 21**).

6. Insufficient representativeness of model protein types. The five selected proteins exclude core CADD research targets such as membrane proteins, multi-subunit complexes, and transmembrane channels, and are all water-soluble single-domain proteins. The model's generalizability to membrane environments and protein-ligand interaction scenarios was not verified, and the dataset should be extended to include diverse protein types.

Author response: We acknowledge that membrane proteins, multi-subunit complexes, and transmembrane channels were not included in the present study. This limitation primarily reflects the scarcity of high-quality experimental datasets for these protein families, particularly deep mutational scanning data.

Nevertheless, we believe that our biophysics-based features remain applicable to more complex systems, as they are designed to capture mutational effects from a biophysical level understanding of protein structure and dynamics. For example, in our previous work [Nordquist et al., Incorporating physics to overcome data scarcity in predictive modeling of protein function: A case study of BK channels. PLOS Computational Biology 19(9): e1011460 (2023)], we applied a random forest model incorporating these biophysics-based features to predict mutational effects on the gating voltage of big potassium (BK) channels using a small dataset (<500 data points). In that study, the model successfully learned the hydrophobic gating mechanism and captured nontrivial voltage-gating properties even in regions with sparse mutational data.

A brief discussion has been added to the concluding Discussion on this point (see **page 21-22**).

7. Unvalidated cross-protein generalization capability. The model was trained separately for each protein (protein-specific training) and did not conduct cross-protein transfer learning experiments (e.g., training on GB1 data to predict avGFP mutations). A core value of AIDD tools lies in cross-target generalization, and cross-protein extrapolation performance data should be added.

Author response: Thank you for this insightful comment. We agree that cross-protein generalization and transfer learning represent an important direction, particularly for extending applicability across diverse targets. In this study, we adopted a protein-specific training strategy to ensure reliable learning from fitness landscapes. Conducting meaningful cross-protein transfer learning experiments would require careful normalization of fitness measurements and a rigorous comparison across datasets. Developing such a global or transferable model remains a challenge even for some state-of-the-art approaches (e.g., Gelman, S. et al. Biophysics-based protein language models for protein engineering. Nat. Methods 22, 1868–1879 (2025)), acknowledging the difficulty of cross-protein generalization observed in recent work on biophysics-based protein language models. Although, it is successful in the case of the MSA-based models such as GVP-MSA [Chen et al., Learning protein fitness landscapes with deep mutational scanning data from multiple sources, Cell Systems 14, 706–721 (2023)]. While these experiments are feasible and highly valuable, a systematic investigation of cross-protein extrapolation is beyond the scope of the current work and will be the focus of future studies.

8. Superficial mechanism analysis for poor performance on highly flexible proteins. The limited performance improvement of Ube4b was attributed to "long-loop structures," but the study did not analyze correlations between RMSF and energy features or quantify the interference of conformational dynamics on mutation effect prediction. Mechanistic research linking protein structure to model performance should be supplemented.

Author response: Thank you for this insightful suggestion. Kindly refer to our response to **Reviewer #1 Point #5** above, where we provide a further analysis of how large-scale conformational dynamics is a main factor that limits the performance for protein Ube4b.

9. Lack of relevance to drug targets. Most selected proteins are tool proteins (e.g., avGFP, GB1), excluding disease-related targets (e.g., kinases, GPCRs). This fails to reflect the model's application value in drug development, and case studies of at least one disease-related target protein should be added.

Author response: Thank you for this valuable comment. We agree that the current study does not include classical drug target proteins, which limits the immediate demonstration of the model's applicability in drug discovery. This limitation primarily arises from the availability of high-quality, suitable datasets for model training and evaluation.

Nevertheless, we have applied our approach to a disease-relevant context related to antibiotic resistance in *Mycobacterium tuberculosis*, where we were successfully able to do the classification of resistance-conferring mutations and sensitive mutations. A manuscript is in late stages of preparation. We agree that incorporating case studies involving well-established disease-related targets would further strengthen the application of the model, and we plan to focus on such targets in future studies as more appropriate datasets become available.
